# The experiences of patients with Duchenne muscular dystrophy in facing and learning about their clinical conditions

**DOI:** 10.3402/qhw.v11.32045

**Published:** 2016-10-05

**Authors:** Haruo Fujino, Yuko Iwata, Toshio Saito, Tsuyoshi Matsumura, Harutoshi Fujimura, Osamu Imura

**Affiliations:** 1 Graduate School of Human Sciences, Osaka University, Suita, Osaka, Japan; 2 Faculty of Education, Oita University, Oita, Japan; 3 Division of Child Neurology, National Hospital Organization Toneyama National Hospital, Toyonaka, Osaka, Japan; 4 Department of Neurology, National Hospital Organization Toneyama National Hospital, Toyonaka, Osaka, Japan

**Keywords:** Duchenne muscular dystrophy, health communication, genetic disease, narration, qualitative study

## Abstract

Patients experience extreme difficulty when facing an intractable genetic disease. Herein, we examine the experiences of patients with Duchenne muscular dystrophy in facing and learning about their disease. A total of seven patients with Duchenne muscular dystrophy (age range: 20–48) participated. We conducted in-depth interviews with them about how they learned of their disease and how their feelings regarding the disease changed over time. Transcribed data were analysed using thematic analysis. The following themes emerged from this analysis: “experiences before receiving the diagnosis,” “experiences when they learned of their condition and progression of the disease,” “supports,” and “desired explanations.” Anxiety and worry were most pronounced when they had to transition to using wheelchairs or respirators due to disease progression; indeed, such transitions affect the patients psychological adjustment. In such times, support from significant others in their lives helped patients adjust.

Duchenne muscular dystrophy (DMD) is an X-linked progressive genetic disease that causes progressive muscle weakness and loss of function. Loss of ambulation and impaired respiratory and cardiac function develop as the disease progresses. Better clinical management, including corticosteroid treatment, non-invasive mechanical ventilation, and cardioprotective medications, has improved prognosis and life expectancy over the last few decades (Bushby et al., 2014; Buyse et al., 2015; Ishikawa et al., 2011; Takeuchi et al., 2013). Although these treatments have proved effective in slowing the decline of muscle function in children, there is currently no curative treatment available.

The life-limiting and progressive nature of the disease strongly affects both patients and family members (Dogba, Rauch, Douglas, & Bedos, 2014; Landfeldt et al., 2014; Read, Kinali, Muntoni, & Garralda, 2010; Read, Kinali, Muntoni, Weaver, & Garralda, 2011; Uzark et al., 2012). Although some parents might tell their child about their condition at a young age, most families do not discuss the possibility of breathing problems or a shortened lifespan with their children (Erby, Rushton, & Geller, 2006) and some avoid telling the child about the condition directly (Imura, 2011). In such cases, children will have limited information about their condition and future trajectories, which can generate a fear of the unknown aspects of their disease. This, in turn, can lead to lower self-esteem and greater anxiety and shame in children (Metcalfe, Plumridge, Coad, Shanks, & Gill, 2011).

Age-appropriate disclosure of a condition can increase patients’ understanding of their disease, which in turn leads to use of better coping strategies and higher well-being in their daily lives (Rowland & Metcalfe, 2013). In contrast, non-disclosure can result in anxiety, guilt, misunderstanding, and higher levels of tension in parents and the affected children. Accordingly, the manner in which patients are informed of their conditions and their own beliefs and attitudes regarding this disclosure can be determinants of their psychological adjustment and acceptance of their conditions (Eiser, Patterson, & Tripp, 1984; Fujino et al., 2015). However, as far as we know, patients’ experiences of the timing of being informed about their diagnosis and the process of becoming conscious of their disease have not been investigated.

In this study, we interviewed adult patients with DMD and asked them to recall their experiences from before and after being told of their diagnosis. Additionally, by examining their experiences of the progression of DMD (e.g., transitioning to use of wheelchair) and what kind of explanation they would have desired from their parents or healthcare providers in retrospect, we aimed to identify better ways of explaining DMD to patients and of providing psychological and emotional support when treating patients with DMD.

## Methods

### Participants

A total of seven patients with DMD participated this study. Five were outpatients and two were inpatients treated at National Hospital Organization Toneyama National Hospital. Their average age was 34.7 years (range: 20–48) (Table I). The criteria for inclusion was as follows: (1) patient with DMD, (2) having ability to answer verbal interview, (3) no sign of mental retardation, and (4) being 20 years of age or older. Most participants had made the transition from walking to using wheelchairs by mid-to-late elementary school. Regarding the use of respirators, although details about the timelines were fuzzy in some cases, more than half of participants (patients A, B, C, and E) had begun using them in high school. At the time of the interview, three participants were using a respirator only at night, two used nasal masks throughout the day, and two had undergone a tracheotomy. All participants, including those who had been receiving care and treatment at home, had been hospitalized in the muscular dystrophy ward of a specialized hospital due to poor health or in order to overhaul the respirator. This study was conducted between October and December 2010. This study was approved by the research ethics committee of the National Hospital Organization Toneyama National Hospital (Ref No. 1011).

**Table I:** Characteristics of the participants.

Patient	Age	Age at diagnosis	Ventilator	Status
A	20	2	NPPV (night)	Outpatient
B	37	7	NPPV (night)	Outpatient
C	35	7	NPPV (night)	Outpatient
D	35	5	NPPV (continuous)	Outpatient
E	38	6	IPPV (tracheotomy)	Outpatient
F	30	9	NPPV (continuous)	Inpatient
G	48	10	IPPV (tracheotomy)	Inpatient

NPPV, non-invasive positive-pressure ventilation; IPPV, intermittent positive-pressure ventilation.

### Procedures

Each semi-structured interview lasted for about 40–70 min for each participant. We recorded the interviews on a digital IC recorder after receiving permission from the participant. During each interview, the interviewer sought information regarding the participant's medical history, developmental history, the moment they learned of their disease, and how their feelings regarding the disease had changed over time; initial probing questions were supplemented by follow-up questions to obtain a deeper understanding of patients’ experiences. Examples of interview questions are shown in Table II.

**Table II:** Sample interview questions.

Before you knew about your illness, did you have thoughts or worries about your body or illness?
How did you find out that you had muscular dystrophy?
What was your understanding of muscular dystrophy at that time?
What did you feel or think about when you learned about your illness?
How did your thoughts and feelings change over time?
What kind of worries did you have when you learned about your diagnosis?
Have you ever consulted anyone on your worries about the disease?
What would be the best way to explain the disease to those diagnosed with the same? What points need to be kept in mind while explaining the diagnosis to patients?

### Data analysis

Qualitative data were analysed using thematic analysis, which is a method of identifying and analysing themes within qualitative data (Braun & Clarke, 2006). First, researchers transcribed the interview data and carefully read several times to familiarize us with the data. Two researchers (HF and YI)—male and female certified clinical psychologists with experience in psychological research in the field of muscular dystrophy—conducted the analysis. Then, each researcher generated initial codes for a short segment of the data. We focused on patients’ experience because this study aimed to examine their experiences of the progression of DMD and desired explanation. After the initial codes were identified, we discussed several times and re-coded the transcripts. Then we created themes based on the coded extracts. Then, we reviewed the themes to confirm whether created themes covered patients’ experience. Disagreements were discussed until consensus was reached.

## Results

### Experiences before learning of their diagnosis and condition

Since the first few years of elementary school—before patients knew anything of their condition and certainly before they had learned about their diagnosis—participants knew that their bodies posed them some problems. They knew they could not participate in certain school activities or be active in the same way as other children their age. However, most patients did not speak of having any emotional responses such as concern or worry related to the condition they were experiencing.

Patient A recalled that he was often late to school or left early from school because he had doctor's appointments. He recalled thinking that it was “exhausting to go to the hospital.” He said, “I am not always astute, so I didn't really worry either. I didn't have any major anxieties.” However, he did realize that his “movements were getting worse.”

Patient C recalled, “Running became harder and I felt how my muscles were getting weaker.” Even with such feelings, since he “didn't know how the disease progresses,” he hardly worried.

Many of the patients spoke of their awareness of the weakening of their muscles and the deterioration of their physical capabilities, but added that they did not think too deeply about the disease itself.

### Experiences of when they learned of their condition and progression of the disease

Each participant reported that their parents or doctors had explained to them something about the disease, but they could not remember the specifics of who said what. Most understood the disease to be something related to their previously noticed decline in muscle strength. Many of the patients could not recall being told clearly what their disease was about, but felt that they had come to understand the condition indirectly through repeated doctor visits, examinations, and rehabilitation activities. Additionally, when visiting specialized hospitals as an outpatient or when they were hospitalized for evaluation, some of the participants observed older patients with DMD, whose disease had progressed further than their own, and realized how their own muscles would weaken over time. One patient reported that he learned about having DMD only after reading a book on the subject, which someone had recommended to him. As they came to know about DMD and their body became less cooperative, their disease became increasingly real to them. On the other hand, Patient F said, “I had some vague understating about the disease when I saw other patients [with DMD] when I was hospitalized. But, I can say that, in fact, I felt relieved on knowing what I had.” Patient D said, “Maybe it is because I didn't quite understand it, but when I learned I had DMD from my parents, I didn't worry too much about it.” Although he had felt the weakening of his muscles, he had a similar reaction when he started using the wheelchair. He reported, “My body was still moving, so I didn't think too much about it. I couldn't think too far ahead.”

Patient E said:

I was in third grade in elementary school when I became aware of my disease. What triggered my awareness was the fact that I could no longer just get up from the bed, as I had used to. I now had to push myself up with my hands. I only came to understand the disease clearly when I stopped walking and became reliant on the wheelchair.

Most patients spoke of how they started to worry or feel anxious when they had to transition to using wheelchairs. Subsequently, as they began using a respirator, they began to wonder how far their disease would progress.

Patient B said that, around the time that walking became a challenge, he often stayed home from school and gradually became dependent on a wheelchair. It was not easy for him to go out because he worried how others looked at him for being in a wheelchair. He said, “I became self-conscious and worried about how others looked at me. Most likely, they didn't care, but I thought they were looking at me.” However, his family began to invite him out more, which gradually lessened these worries. He also stated that he could truly feel the progression of the disease when he started using a respirator.

For Patient E, the most difficult time came when he transitioned to using a wheelchair, which was in elementary school. He was bullied during that time, and recalled thinking that:

They would not have bullied me if I did not have this disease. Why did I get this disease? I mean, at that time, we were kids in third grade, and we really didn't know anything. I think they bullied me because after they had helped carry me in my wheelchair all the way up to the classrooms on the second or third floor, I failed to say “thank you,” so they took offense. So, they just said, “what the …” and bullied me. That was the most difficult time for me.

However, he mentioned that, in higher grades, “others started to treat me better, and I felt that they had finally gained an understanding. That brought me comfort. I think it made it emotionally much easier on me.”

Some of the patients expressed a strong resistance towards using a respirator, and spoke of the fact that they were shocked at the idea of needing to use one. Patient A recognized the progression of the disease when his muscles weakened to the point that he needed to use a respirator. He thought to himself, “My body's condition has changed … Will I have to wear [the respirator] during the day, too?” He explained, “I started to worry about how much more my functions would become restricted. No, it felt more like panic.” That gave him a sense of urgency and he began to focus on rehabilitation more than ever before. “There were things like my hands feeling weaker when opening them, and I wanted to see if I could at least slow the progression before it became any harder to move my body.”

When Patient C was in high school, his doctor recommended that he meet another patient who had undergone a tracheotomy.

When I saw him, he was sleeping, and all he had going for him was his respirator. I thought I would rather die than ever become like that. I remember telling my mother to take it off if I ever become like that.

Patient D said about the decision to begin using a respirator, “I was a bit shocked as I had no idea that we had to put such a machine [i.e., the respirator] on.” After being hospitalized, he said “there were other people with muscular dystrophy and some were using respirators, so when I saw that, over time, well, I kind of accepted it.”

### Supports

All the patients said that they had never discussed their concern about the disease with their parents or other family members. The following reasons were cited for not consulting their family: “No point in thinking about it,” “the disease didn't bother me,” and “I did not want to worry my parents.” Some patients reported that they discussed their physical condition or what type of wheelchairs they used with other friends who had DMD.

### Desired explanations

Regarding what patients believed would be the ideal time or way to have their conditions explained to them, although each participant had several ideas, the most commonly reported was that it is best to inform patients when the disease progression resulted in a change in their overall condition, such as necessitating the transition to a wheelchair. Some patients also said that they would prefer to discuss their disease in the absence of their parents.

Patient A said, “When there is no way to cure the disease, it's not good to give false hope.” He wanted newly diagnosed patients to know that it is good to have hope, but added that when the disease progresses and their condition deteriorate, “you might be too devastated, and that shock could be quite bad. So, it's better not to have too much hope either.” He preferred to be told about the disease as changes took place. He said,

Each person's condition and the way they think about their disease are different. I do not want people to bundle all DMD patients as if they are all the same. I want to have people look at each person as an individual.

He also advised:

I want you to find something you love so much that you forget all your fears and worries about the disease, your own body's condition, or the changes the disease brings. Find what things you like to do, follow your interest, and lose yourself in it. Enjoy life. This is how you should live.

As for the points to consider while explaining the disease, Patient C said, “Kids tend to look at the expressions of their parents. It might be difficult for them (the kids) to talk about it.” Moreover, he added:

I think that rather than talking to the kid alone, maybe it would be good to have another kid with the same kind of disability. I think the parents should not be there at that time … Of course, whether it is a parent or a doctor, when asked, they should just answer directly.

He then added that if the patient does not want to know about the disease or the diagnosis, it is recommended that others wait until this patient is ready to hear it. However, he said, “you should at least tell them that their muscles will get weaker.” Patient C was also aware of how his mobility decreased as the disease progressed, particularly when he began needing to use a respirator. Therefore, he wanted to advise other children to “play” before their disease worsens to the point where they need a respirator. He also added that young children, although they might not understand it, must be told that “there are plenty of people out there that don't give up and have fun despite their disease.”

Patient D said, “I wish I knew more about the whole thing (including the prognosis) much sooner.” He added that if he had known the same, he might have put more effort into rehabilitation. He felt that patients must be told that they would at some point need to rely on wheelchairs. As for the disease itself, he said, “I think you should tell them the whole truth. They might be shocked by it, but it's better to tell them.”

Finally, Patient F said:

About informing the patient about the diagnosis, you just need to look at the individual situation. Parents should not hide the disease from their children. They would be too shocked if they were told too late. It is better to tell them early on. But, all of this is not easy to understand as a kid, especially regarding what happens after they start using the wheelchairs. Therefore, rather than explaining to them about the disease, it might be better to show them the condition of other patients.

## Discussion

In this study, we examined DMD patients’ experiences about knowing about their diagnosis. Most patients did not feel scared or anxious about their condition before learning about their disease. However, transitioning to a wheelchair and respirator had considerable impacts on them. In the process, seeing other patients who had undergone the same was a crucial experience for them.

When patients with DMD receive their official diagnosis, often, only the parent actually receives an explanation from the doctor (Imura, 2011). This means that young patients do not often learn the name of the disease or receive an adequate explanation about their condition (Takada & Imura, 2011). In the present study, even before patients learned of the name of their disease or condition, most patients reported that they were aware of their declining muscle strength. In a previous study, parents had an important role in telling their children about their disease; however, in half of the families of this previous study, parents did not tell their children the name of the disease, while some parents only told them that they had DMD (Plumridge, Metcalfe, Coad, & Gill, 2010).

Parents of patients with DMD, especially mothers, often find it difficult to share information due to their own emotional pain and sense of guilt about DMD (Plumridge et al., 2010). Even when they suspected that their children knew about their poor prognosis or other aspects of their disease, many parents avoided saying anything to their children until they were directly asked by the child (Erby et al., 2006). Accordingly, children with DMD have little information in comparison with children with other genetic conditions (Plumridge et al., 2010).

The patients experienced more worry and fear when they transitioned to using a wheelchair or a respirator. The worries and fears related to how their condition would continue to worsen, how their quality of life would change with the decline in their ability to do things on their own, and how others might view them because they used a wheelchair to get around. It is possible that their fears and worries could be alleviated through the support of friends and family in helping patients better adjust to these changes (Pehler & Craft-Rosenberg, 2009).

Similarly, the use of a respirator was viewed by some of the participants as stigmatizing. Indeed, its use conjured up images of being tied up to machines, which has been reported in previous studies as well (Miller, Colbert, & Schock, 1988). Regarding the use of a respirator at home, the following may be sources of potential distress for patients: dependence on others, a lack of understanding by the people around them, difficulty in going out, and worries related to aging parents who are their primary caregivers at home (Van Kesteren, Velthuis, & Van Leyden, 2001). As such, it seems necessary to help patients adjust during this period, as it could improve their quality of life after introduction of the respirator (Abbott & Carpenter, 2015).

Support groups such as patients associations can lend emotional and psychological support to both patients and parents (Hodges & Dibb, 2010; Plumridge et al., 2010). Those activities are also an opportunity for patients to see others with the same disease but who might be at a more advanced stage, although those could be a point of resistance for some children (Abbott & Carpenter, 2015; Erby et al., 2006; Firth, Gardner-Medwin, Hosking, & Wilkinson, 1983).

In general, desired explanation about the disease depends on patients’ characteristics, including patients’ age, comprehension, and disease condition (Fujino, Saito, Imura, Matsumura, & Shinno, 2013). Most participants preferred that they could obtain information about the nature of DMD in earlier age although these results may be affected by the characteristics of the participants (i.e., age). Open communication could help children to be emotionally resilient while parents may feel difficult and emotional pain in sharing the information with their children (Metcalfe, Coad, Plumridge, Gill, & Farndon, 2008). In contrast, thinking of living with DMD could be a burden for young patients (Abbott, Carpenter, & Bushby, 2012). The progressive nature of DMD may be an important factor that affects patients’ future prospects and amount of information explained by parents and doctors (Fujino et al., 2013). We noted that, rather than seeking out any explanation, most patients observed and spoke to older patients during hospital visits or when they were hospitalized, which had a greater impact on them than what their parents told them. They may shape their understanding of their disease by observing other patients using a wheelchair or respirator or those who were bedridden.

By nature, DMD is a slowly progressing disease. However, our results are clear in showing that transitioning to a wheelchair or a respirator can be a trigger for anxiety and worry; therefore, it would be beneficial to provide special attention and assistance to patients with DMD during this phase. Furthermore, DMD patients are known to have a greater risk of developmental and psychological problems (Banihani et al., 2015; Hinton et al., 2009; Ricotti et al., 2015; Steele et al., 2008). Therefore, it would be prudent to provide support in that area as well (Yamaguchi & Suzuki, 2015). Although patients experienced worry and anxiety in facing changes relating to the progression of DMD—perhaps because they did not want to face their current selves with the disease, or their future selves with a more advanced state—they did not reach out for information or support. Thus, further study on how to promote such reaching out is necessary.

### Limitations

This study has several limitations. We recruited participants from a hospital. It is possible that the results may be skewed or biased in one direction or another. Only seven patients were interviewed in this study because no new theme was identified after the fifth patient's interview. Additionally, this study had some methodological limitations. As the study essentially involved patients recalling their memories in an interview, it is possible that what the patients recalled was factually different from what actually occurred. Their thoughts and needs may be different to when they were younger. Future studies could consider focusing on patients still in their youth to eliminate this possibility. Further, with regard to how patients learned of their disease, it would be important to clarify parents’ perspectives on how they relate to and communicate disease-related information to their children with DMD. Furthermore, ways of communicating the diagnosis in the family could differ by location and cultural background (Fitzpatrick & Barry, 1990). By studying interactions among family members, the experiences of the patient and the family will become clearer. Therefore, future studies should explore these aspects.

## Conclusion

In this study, we clarified the experiences of adult patients with DMD regarding how they came to know about their disease by examining their recollections. Most patients shared how transitioning to the use of a wheelchair or respirator triggered anxiety and worry. Furthermore, the support of significant others in their lives helped the patients adjust to their changing reality.
